# The frequency of class1 and 2 integrons in *Pseudomonas aeruginosa* strains isolated from burn patients in a burn center of Ahvaz, Iran

**DOI:** 10.1371/journal.pone.0183061

**Published:** 2017-08-15

**Authors:** Azar Dokht Khosravi, Moloudsadat Motahar, Effat Abbasi Montazeri

**Affiliations:** 1 Infectious and Tropical Diseases Research Center, Health Research Institute, Ahvaz Jundishapur University of Medical Sciences, Ahvaz, Iran; 2 Department of Microbiology, School of Medicine, Ahvaz Jundishapur University of Medical Sciences, Ahvaz, Iran; Centre National de la Recherche Scientifique, FRANCE

## Abstract

**Background:**

*Pseudomonas aeruginosa* is an opportunistic pathogen with the ability to cause severe nosocomial infections and remains a major problem in burn patients. This organism shows a remarkable antimicrobial resistance and is often resistant to multiple antibiotics. Integron genes as mobile genetic elements are playing an important role in the spread of *P*. *aeruginosa* antibiotic resistance. This study was aimed to investigate the occurrence of class 1, and 2 integron genes (*int1*, *int2)*, among *P*. *aeruginosa* strains isolated from patients with burn infections.

**Methods:**

In total 93 clinical isolates of *P*. *aeruginosa* were screened. The antimicrobial susceptibilities of 9 common antimicrobial agents were tested against the isolates using disk diffusion method. PCR amplification was performed on extracted DNAs for the detection of *int1*, and *int2* genes using the set of specific primers.

**Results:**

The majority of *P*. *aeruginosa* isolates were from wound infection (69.9%). In disk diffusion method, most isolates showed remarkable resistance to tested antibiotics with highest against gentamicin (94.62%) and ciprofloxacin (93.55%). PCR amplification revealed that 89(95.7%) of *P*. *aeruginosa* strains carried *int1*, but none of them harbored *int2* genes. The distribution of *int1* gene was highest in blood (100%), followed by wound isolates (95.38%).

**Conclusions:**

We demonstrated a high antimicrobial resistance among *P*. *aeruginosa* isolates in our setting. *int1* was prevalent and seems to play an important role in multidrug resistance among the isolates. So, performance of antibiotic surveillance programs is necessary for choosing the appropriate therapy and management of infection control practices.

## Introduction

*Pseudomonas aeruginosa* is an opportunistic pathogen with the potential to cause severe healthcare-associated infections especially in immune-compromised patients suffering from AIDS, cancer, burn wounds and respiratory infections such as cystic fibrosis [1, 2]. In general, burn wounds through suppression of immune system provide a suitable site for bacterial multiplication, therefore, *P*. *aeruginosa* infection in burn patients is common and accounts as one of the most serious life threatening conditions in burn units [3]. Moreover, because of the resistance of these microorganisms to a wide variety of antibiotics in recent years, treatment of infections caused by them has been difficult and leads to increased mortality [4]. The difficulty of eradication of *P*. *aeruginosa* infection is due to its intrinsic resistance to different antibiotics caused by several mechanisms including low outer membrane permeability, overexpression of efflux pump system and enzymatic antibiotic modifications e.g. β-lactamase production [5].

Dissemination of antibiotic resistance genes by mobile genetic elements is an increasing concern worldwide which can cause the evolution of multi-drug resistant (MDR) strains among clinical isolates [6].

Integrons are genetic elements which linked to transposons, plasmids and chromosome. These are responsible for development of bacterial resistance in particular among Gram-negative bacterial pathogens [7, 8]. Integrons compose of three essential core elements: The *intI* gene encodes an integrase (*IntI*), needed for site-specific recombination; *attI*, the adjacent recombination site which is recognized by integrase; and integrin associated promoter (Pc), that is required for transcription and expression of gene cassettes within the integron. Gene cassettes are genetic elements that encode antibiotic resistance genes, and consist of a specific-site recombination recognized by integrase that called *attC* [or 59-base elements] [9].

Based on the differences in the amino acid sequences of encoded integrases, integrons have been divided into five classes [10]. The class 1 integron with the greatest diversity of gene cassettes, is the most clinically important, and has a wide distribution among resistant strains [11]. Due to the importance of integrons in the spread of antibiotic resistance, the present study was aimed to investigate the occurrence of class 1, and 2 integrons among *P*. *aeruginosa* strains isolated from patients with burn infections in Ahvaz, Iran.

## Materials and methods

### Bacterial isolates

A total of 93 isolates of *P*. *aeruginosa* were collected from different clinical specimens (blood, urine, wound, etc.), at the laboratory of Taleghani Burn Hospital of Ahvaz, from March 2016 to August 2016. According to the laboratory information, each isolate was belonged to a separate patient. The study was approved by Institutional Review Board (IRB) and Ethics of the Ahvaz Jundishapur University of Medical Sciences, after submission of preliminary proposal, and necessary permission for sample collection was granted. Though the hospital laboratory provided the isolates as *P*. *aeruginosa*, however for double check, all isolates were re-identified by using standard culture method and confirmed by Gram staining and routine biochemical tests including Oxidase test, Oxidative-Fermentative test, growth on Cetrimide agar, growth at 42°C, and pigment production in Mueller Hinton agar (Merck, Germany) [12].

### Drug Susceptibility Testing (DST)

DST was performed using disk diffusion method (Kirby-Bauer) on Mueller-Hinton agar (Merck, Germany) plates according to the Clinical and Laboratory Standards Institute (CLSI) guideline [13]. The tested antibiotic disks were: imipenem (10μg), meropenem (10μg), amikacin (30μg), ciprofloxacin (5μg), ceftriaxone(30μg), ceftazidime (30μg), colistin-sulfate (10μg), pipracillin-tazobactam (100/10μg), and gentamicin (10μg) [Mast Co., UK]. *P*. *aeruginosa* ATCC 27853 was used as a control strain for DST.

### DNA extraction and PCR amplification

DNA was extracted from colonies of *P*. *aeruginosa* isolates by simple boiling method as described elsewhere [14]. In brief, a few colonies were removed from fresh overnight culture on Muller- Hinton agar plates and dissolved in 500 μl of TE buffer, boiled for 10 min and placed at -20°C for 5 min. After centrifugation at 14000 × g for 10 min, the supernatant was used as a template for PCR.

### Detection of class 1, and 2 integrons by multiplex Polymerase Chain Reaction (PCR)

PCR amplification was performed on extracted DNAs for the detection of *int1*, and *int2*, genes using the set of primers described previously and are listed in Table 1 [15].

**Table 1:** Sequences of primers used for detection of integrase genes.

Target gene	Primer sequence(5´→3´)	Size(bp)	Annealing Temprature (°C)
***int1*-F**	CAGTGGACATAAGCCTGTTC	160	59
***int1*-R**	CCCGAGGCATAGACTGTA		
***int2*-F**	CACGGATATGCGACAAAAAGGT	789	59
***int2*-R**	GTAGCAAACGAGTGACGAAATG		

PCR mix was prepared in a final volume of 25 μl containing 10X PCR Buffer, MgCl2 50 mM, dNTPs 10 mM, each primer 10 μM, Taq DNA Polymerase 5 U/μl, and 5 μl of extracted DNA. The amplification was performed in a thermocycler (Eppendorf, Germany), with following program: initial denaturation at 94°C for 5 min, 30 cycles of denaturation at 94°C for 1 min, annealing at 59°C for 1 min, extension at 72°C for 1 min and a final extension at 72°C for 5 min. *P*. *aeruginosa* ATCC 27853 was used as positive control.

The PCR products were separated by electrophoresis on a 1.5% agarose gel containing 0.5 μg/ml ethidium bromide. The bands were visualized under UV light using a gel documentation system (Proteinsimple, USA).

Descriptive statistics including frequencies, cross-tabulation of microbiological, clinical and demographic data were analyzed using SPSS statistical software (version 22.0). The Chi square test, was used in a univariate analysis to assess the differences between two groups of isolates (integron positive and integron negative) with antibiotic resistance. *p* values less than 0.05 were considered statistically significant.

## Results

In the present study, 93 *P*. *aeruginosa* isolates from burn infections were screened in view of antibiotic resistance and presence of *int1*& *int2* genes. The distribution of *P*. *aeruginosa* isolates in different clinical specimens were as follows: wound, 65(69.9%); blood, 14(15.05%); burn tissue biopsy, 7(7.53%); urine, 4(4.3%); and stool, ear discharge, and catheter one each (1.08%). Forty-one (44.09%) isolates were from male and 52(55.91%) were from female patients. The origin of *P*. *aeruginosa* isolates according to the hospital department are presented in Table 2. Intensive care unit with 48 isolates (51.61%), and Pediatrics with 3 isolates (3.23%), comprised the highest and lowest rates of *P*. *aeruginosa* isolation respectively.

**Table 2:** Prevalence of *P*. *aeruginosa* isolates in different hospital departments according to the type of clinical samples.

	Sample No. (%)						
	Wound	Blood	Biopsy	Urine	Stool	Ear	Catheter
ICU	33(35.48)	10(10.75)	4(4.3)	-	-	-	1(1.08)
Internal (W)	16(17.2)	3(3.23)	3(3.23)	-	-	-	-
Plastic surgery	8(8.6)	1(1.08)	-	1(1.08)	1(1.08)	1(1.08)	-
Internal(M)	5(5.38)	-	-	3(3.23)	-	-	-
Pediatrics	3(3.23)	-	-	-	-	-	-

ICU: Intensive Care Unit; W: women; M: men

**Fig 1:**
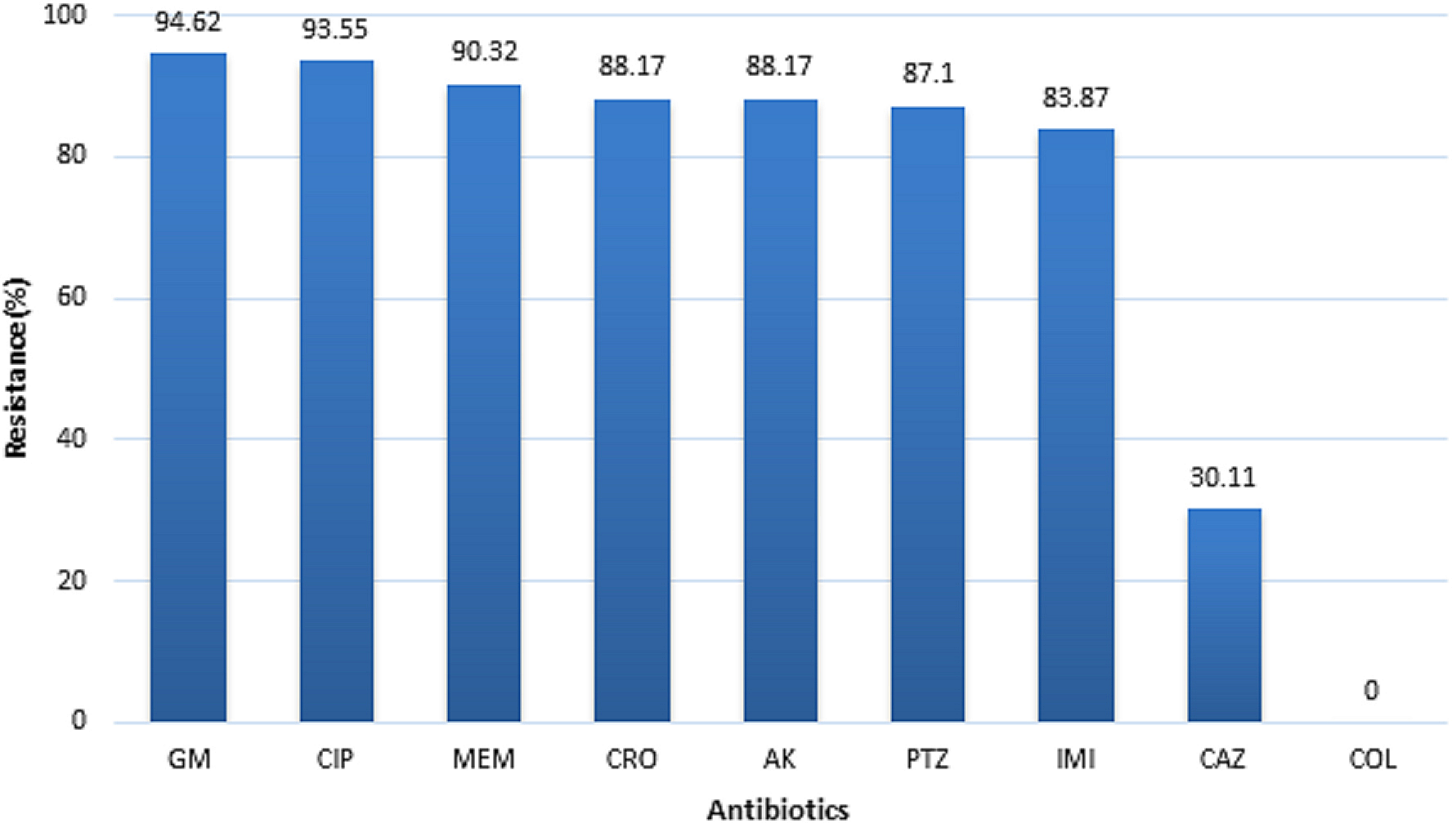
Antimicrobial resistance pattern of *P*. *aeruginosa* isolates. GM: Gentamicin, CIP: Ciprofloxacin, MEM: Meropenem, CRO: ceftriaxone; AK: Amikacin, PTZ: Pipracillin/Tazobactam, IMI: Imipenem, CAZ: ceftazidime; COL: Colistin.

On the basis of the obtained results from DST, most isolates showed high resistance to gentamicin (94.62%) and ciprofloxacin (93.55%). However, the isolates were fully susceptible to colistin (100%). Resistance pattern of *P*. *aeruginosa* isolates against 9 tested antibiotics is shown in Fig 1.

Based on the PCR results, 89 (95.7%) of *P*. *aeruginosa* strains carried *int1*gene, but none of the isolates harbored *int2* gene. The distribution of *int1* gene among the isolates in relation to originated clinical samples was highest for blood (100%), followed by wound (95.38%).

In Table 3, the relationship between *P*. *aeruginosa* strains comprising *int1* and antibiotic resistance are presented. While all resistant strains harbored the gene, some susceptible strains contained the gene as well.

**Table 3:** Association between presence of integron and antibiotic resistance among 93 *P*. *aeruginosa* clinical isolates.

Antibiotics		Integron positive isolates (n = 89)			Integron-negative isolates (n = 4)		P value
	R No. (%)	I No. (%)	S No. (%)	R No. (%)	I No. (%)	S (%)	
**GM**	88(98.88)	0	1(1.12)	0	0	4(100)	0.001
**CIP**	87(97.75)	0	2(2.25)	0	0	4(100)	0.001
**MEM**	84(94.38)	1(1.12)	4(4.49)	0	0	4(100)	0.001
**CRO**	82(92.13)	2(2.25)	5(5.62)	0	0	4(100)	0.001
**AK**	82(92.13)	1(1.12)	6(6.74)	0	0	4(100)	0.001
**PTZ**	81(91.01)	3(3.37)	5(5.62)	0	0	4(100)	0.001
**IMI**	78(87.64)	5(5.62)	6(6.74)	0	0	4(100)	0.001
**CAZ**	28(31.46)	3(3.37)	58(65.17)	0	0	4(100)	0.352
**COL**	0	0	89(100)	0	0	4(100)	NS

GM:gentamicin; CIP: ciprofloxacin; MEM: meropenem; CRO: ceftriaxone; AK: amikacin; PTZ: Pipracillin/tazobactam; IMI: imipenem; CAZ: ceftazidime; COL: colistin. R: resistant; I: intermediate; S:sensitive; NS: not statistically significant

## Discussion

The spread of antibiotic resistance genes among bacteria including *P*. *aeruginosa* strains is an increasing concern in treatment of nosocomial infections. Many resistance genes are present as gene cassettes within integrons, which may themselves be located on transmissible plasmids and transposons [15].

In this study, according to DST, most of our isolates showed high resistance (>80%) to antimicrobial agents tested except for colistin with the susceptibility rate of 100%. A previous study conducted by Khosravi & Mihani in the same region [16], lower resistance was reported for most tested antibiotics. Among them, a resistance rate of 41% were demonstrated for both imipenem and meropenem, while our study showed a higher resistance to carbapenem antibiotics which shows an increasing trend of carbapenem resistance in burn patients in Ahvaz. Disconcordant to our study, a low resistance level was reported for imipenem (23.3%) in northern province of Guilan in Iran [17]. Additionally, the resistance rate of our isolates against aminoglycoside groups; gentamicin and amikacin were 94.62% and 88.17% respectively. While in agreement to our findings, high resistance for aminoglycoside antibiotics were reported by Poonsuk et al. [18], the lower rates of resistance to these antibiotics were also demonstrated by other investigators [19–21]. Moreover, the resistance rate to ceftriaxone and ceftazidime were 88.17% and 30.11% in this study respectively, which was in concordant to finding of Odumosu et al. with reported rates of 87.1% and 22.5% for mentioned antibiotics [22].

In the present study, PCR amplification for screening of the presence of two classes of integrase genes, showed that 89 *P*. *aeruginosa* isolates (95.7%), were contained *int1* gene. The prevalence of *int1* gene in clinical isolates of *P*. *aeruginosa*, is reported differently in similar investigations from Iran ranged from 43% to 56.3% [17, 23], and around the world [6, 15, 24]. In mentioned reports and other documented studies, the prevalence is lower compared to the present study and this could be due to a trend of rapid increasing of integron positive rate among clinical isolates of *P*. *aeruginosa* in our region in recent years.

According to the definition for multidrug resistance (MDR) demonstrating resistance to at least three of six drugs, including amikacin, gentamicin, ciprofloxacin, piperacillin, ceftazidime and imipenem [25], all the isolates in our setting were shown to be MDR. Integrons are known to be associated with MDR, especially class 1 integrons, which are widely distributed in Gram-negative bacteria including *P*. *aeruginosa* [15]. We found high antimicrobial resistance among isolates that were positive for *int1* gene, mainly for gentamicin (94.62%), ciprofloxacin (93.54%), and meropenem (90.32%). Chen et al., reported the rates of 90.1% and 88.7% for ceftazidime and ceftriaxone respectively among their *int1* gene positive isolates [26]. Though the resistance rate for ceftriaxone was similar to our finding, however, we demonstrated a lower level of resistance for ceftazidime (30.11%). As mentioned earlier, investigators documented that MDR correlated strongly with the presence of integrons, and the majority of our MDR isolates comprised *int1* gene, however, there were 4 MDR isolates with no integrons, and therefore other factors should be noted that make these bacteria resistant to antibiotics.

Furthermore, our finding revealed the presence of *int1* gene in some isolates sensitive to certain antibiotic, notably 6 integron-comprising isolates that were sensitive to amikacin and 5 susceptible isolates to ceftriaxone and pipracillin/tazobactam (Table 3).

While the prevalence of *int2* gene is documented in some reports [24, 27], none of *P*. *aeruginosa* strains in this study were found to carry *int2* gene, which was in concordant to other previous reports [6, 25].

According to the results from the present study, it seems that most antibiotics used in our investigation are inappropriate drugs for the treatment of *P*. *aeruginosa* infections. Among them, colistin followed by ceftazidime were the most sensitive antibiotics and could be the drugs of choice. The susceptibility of all the isolates to colistin may be explained by the low application of this antibiotic in routine treatments of burn patients.

In conclusion, we demonstrated a high antimicrobial resistance among *P*. *aeruginosa* isolates in our setting. *int1* gene was prevalent and seems to play an important role in multidrug resistance among the isolates. So, performance of antibiotic surveillance programs is necessary for choosing the appropriate therapy and management of infection control practices.

## References

[1] Mesaros N, Nordmann P, Plésiat P, Roussel‐Delvallez M, Van Eldere J, Glupczynski Y, et al *Pseudomonas aeruginosa*: resistance and therapeutic options at the turn of the new millennium. Clin Microbiol Infect. 2007;13(6):560–78. doi: 10.1111/j.1469-0691.2007.01681.x 17266725 10.1111/j.1469-0691.2007.01681.x

[2] Strateva T, Yordanov D. Pseudomonas aeruginosa–a phenomenon of bacterial resistance. J Med Microbiol. 2009;58(Pt 9):1133–48. doi: 10.1099/jmm.0.009142-0 19528173 10.1099/jmm.0.009142-0

[3] Church D, Elsayed S, Reid O, Winston B, Lindsay R. Burn wound infections. Clin Microbiol Rev. 2006;19(2):403–34. doi: 10.1128/CMR.19.2.403-434.2006 16614255 10.1128/CMR.19.2.403-434.2006 PMC1471990

[4] Fair RJ, Tor Y. Antibiotics and bacterial resistance in the 21st century. Perspect Medicin Chem. 2014;6:25–64. doi: 10.4137/PMC.S14459 25232278 10.4137/PMC.S14459 PMC4159373

[5] Breidenstein EB, de la Fuente-Núñez C, Hancock RE. *Pseudomonas aeruginosa*: all roads lead to resistance. Trends Microbiol. 2011;19(8):419–26. doi: 10.1016/j.tim.2011.04.005 21664819 10.1016/j.tim.2011.04.005

[6] Sun G, Yi M, Shao C, Ma J, Zhang Q, Shao S. Novel Class 1 Integrons in multi-drug resistant isolates from Eastern China. Indian J Microbiol. 2014;54(2):227–31. doi: 10.1007/s12088-013-0441-9 25320427 10.1007/s12088-013-0441-9 PMC4188487

[7] Fluit A, Schmitz FJ. Resistance integrons and super‐integrons. Clin Microbiol Infect. 2004;10(4):272–88. doi: 10.1111/j.1198-743X.2004.00858.x 15059115 10.1111/j.1198-743X.2004.00858.x

[8] Gillings MR. Integrons: past, present, and future. Microbiol Mol Biol Rev. 2014;78(2):257–77. doi: 10.1128/MMBR.00056-13 24847022 10.1128/MMBR.00056-13 PMC4054258

[9] Domingues S, da Silva GJ, Nielsen KM. Integrons: vehicles and pathways for horizontal dissemination in bacteria. Mob Genet Elements. 2012; 2(5):211–223. doi: 10.4161/mge.22967 23550063 10.4161/mge.22967 PMC3575428

[10] Cambray G, Guerout AM, Mazel D. Integrons. Annu Rev Genet. 2010;44:141–66. doi: 10.1146/annurev-genet-102209-163504 20707672 10.1146/annurev-genet-102209-163504

[11] Deng Y, Bao X, Ji L, Chen L, Liu J, Miao J, et al Resistance integrons: class 1, 2 and 3 integrons. Ann Clin Microbiol Antimicrob. 2015;14:45 doi: 10.1186/s12941-015-0100-6 26487554 10.1186/s12941-015-0100-6 PMC4618277

[12] Hall GS. Bailey & Scott’s Diagnostic Microbiology. The Oxford University Press; 2013.

[13] Patel J, Cockerill F, Alder J, Bradford P, Eliopoulos G, Hardy D. Performance standards for antimicrobial susceptibility testing; twenty-fourth informational supplement. CLSI standards for antimicrobial susceptibility testing 2014;34:1–226.

[14] Lee S, Park Y-J, Kim M, Lee HK, Han K, Kang CS, et al Prevalence of Ambler class A and D β-lactamases among clinical isolates of *Pseudomonas aeruginosa* in Korea. J Antimicrob Chemother 2005; 56: 122–7. doi: 10.1093/jac/dki160 15890715 10.1093/jac/dki160

[15] Gu B, Tong M, Zhao W, Liu G, Ning M, Pan S, et al Prevalence and characterization of class I integrons among *Pseudomonas aeruginosa* and *Acinetobacter baumannii* isolates from patients in Nanjing, China. J Clin Microbiol. 2007;45(1):241–3. doi: 10.1128/JCM.01318-06 17122024 10.1128/JCM.01318-06 PMC1828976

[16] Khosravi AD, Mihani F. Detection of metallo-β-lactamase–producing *Pseudomonas aeruginosa* strains isolated from burn patients in Ahwaz, Iran. Diagn Microbiol Infect Dis. 2008; 60(1):125–8. doi: 10.1016/j.diagmicrobio.2007.08.003 17900848 10.1016/j.diagmicrobio.2007.08.003

[17] Nikokar I, Tishayar A, Flakiyan Z, Alijani K, Rehana-Banisaeed S, Hossinpour M, et al Antibiotic resistance and frequency of class 1 integrons among *Pseudomonas aeruginosa*, isolated from burn patients in Guilan, Iran. Iran J Microbiol. 2013; 5(1):36–41. 23466812 PMC3577559

[18] Poonsuk K, Tribuddharat C, Chuanchuen R. Class 1 integrons in *Pseudomonas aeruginosa* and *Acinetobacter baumannii* isolated from clinical isolates. Southeast Asian J Trop Med Public Health. 2012; 43(2):376–84. 23082590

[19] Moosavian M, Rahimzadeh M. Molecular detection of metallo-β-lactamase genes, *blaIMP-1*, *blaVIM-2* and *blaSPM-1* in imipenem resistant *Pseudomonas aeruginosa* isolated from clinical specimens in teaching hospitals of Ahvaz, Iran. Iran J Microbiol. 2015;7(1):2–6. 26644866 PMC4670463

[20] Sheikh AF, Rostami S, Jolodar A, Tabatabaiefar MA, Khorvash F, Saki A, et al Detection of metallo-Beta lactamases among carbapenem-resistant *Pseudomonas aeruginosa*. Jundishapur J Microbiol. 2014;7(11):e12289 doi: 10.5812/jjm.12289 25774271 10.5812/jjm.12289 PMC4332233

[21] Cicek AC, Saral A, Duzgun AO, Cizmeci Z, Kayman T, Balci PO, et al Screening of Class 1 and Class 2 integrons in clinical isolates of *Pseudomonas aeruginosa* collected from seven hospitals in Turkey: A multicenter study. Open J MedMicrobiol 2013;3:277–33.

[22] Odumosu BT, Adeniyi BA, Chandra R. Analysis of integrons and associated gene cassettes in clinical isolates of multidrug resistant *Pseudomonas aeruginosa* from Southwest Nigeria. Ann Clin Microbiol Antimicrob. 2013; 12:29 doi: 10.1186/1476-0711-12-29 24143920 10.1186/1476-0711-12-29 PMC3842740

[23] Yousefi S, Nahaei M, Farajnia S, Ghojazadeh M, Akhi M, Sharifi Y, et al Class 1 integron and imipenem resistance in clinical isolates of *Pseudomonas aeruginosa*: prevalence and antibiotic susceptibility. Iran J Microbiol. 2010;2(3):115–21. 22347559 PMC3279778

[24] Xu Z, Li L, Shirtliff ME, Alam M, Yamasaki S, Shi L. Occurrence and characteristics of class 1 and 2 integrons in *Pseudomonas aeruginosa* isolates from patients in Southern China. J Clin Microbiol. 2009;47(1):230–4. doi: 10.1128/JCM.02027-08 19020065 10.1128/JCM.02027-08 PMC2620863

[25] Rossolini G, Mantengoli E. Treatment and control of severe infections caused by multiresistant *Pseudomonas aeruginosa*. Clin Microbiol Infect. 2005 7;11 Suppl 4:17–32. 10.1111/j.1469-0691.2005.01161.x 15953020

[26] Chen J, Su Z, Liu Y, Wang S, Dai X, Li Y, et al Identification and characterization of class 1 integrons among *Pseudomonas aeruginosa* isolates from patients in Zhenjiang, China. Int J Infect Dis 2009, 13:717–721. doi: 10.1016/j.ijid.2008.11.014 19208492 10.1016/j.ijid.2008.11.014

[27] Goudarzi M, Fazeli M, Azad M, Seyedjavadi S, Mousavi R, Rashidan M, et al Carriage of class 1 and class 2 Integron in multidrug resistant *Pseudomonas aeruginosa* isolated from burn patients in Tehran hospitals, Iran. West Indian Med J. 2015; 65(1):32–39. doi: 10.7727/wimj.2014.315 26633135 10.7727/wimj.2014.315

